# HBx increases chromatin accessibility and ETV4 expression to regulate dishevelled-2 and promote HCC progression

**DOI:** 10.1038/s41419-022-04563-9

**Published:** 2022-02-04

**Authors:** Chuqian Zheng, Min Liu, Yanping Ge, Yanyan Qian, Hong Fan

**Affiliations:** 1 Department of Medical Genetics and Developmental Biology, School of Medicine, The Key Laboratory of Developmental Genes and Human Diseases, Ministry of Education, Southeast University, Nanjing, China; 2 School of Life Science and Technology, Southeast University, Nanjing, China

**Keywords:** Cancer epigenetics, Cell invasion

## Abstract

Hepatitis B virus (HBV) infection is the predominant causes of hepatocellular carcinoma (HCC). HBV X protein (HBx), as the most frequently integrated viral gene sequence following HBV infection, plays a critical role in the pathogenesis of HCC. H3K27ac is a characteristic marker for identifying active enhancers and even indicates chromatin accessibility associated with super-enhancers (SEs). In this study, H3K27ac ChIP-seq was applied for high-quality SE annotation of HBx-induced SEs and chromatin accessibility evaluation. The results indicated that HBx preferentially affects enrichment of H3K27ac in transcription factor signaling pathway genes, including ETV4. RNA-seq indicated that ETV4 is upregulated by HBx and that upregulated ETV4 promotes HCC progression. Interestingly, ETV4 was also included in the 568 cancer driver gene pool obtained by the Integrative OncoGenomics pipeline. However, the biological function and mechanism of ETV4 remain incompletely understood. In vivo and in vitro, we found that increased ETV4 expression promotes HCC cell migration and invasion by upregulating DVL2 and activating Wnt/β-catenin. The mRNA and protein levels of ETV4 are higher in tumor tissues compared with adjacent tissues, and high expression of ETV4 is associated with poor prognosis in HCC patients. In summary, we first confirm that ETV4 is significantly upregulated by HBx and involved in SE-associated chromatin accessibility. Increased expression of ETV4 promotes HCC cell invasion and metastasis by upregulating DVL2. The present study provides insight into the ETV4-DVL2-β-catenin axis in HBV-related HCC, which will be helpful for treating patients with aggressive HCC.

## Introduction

Liver cancer is recognized as the sixth most common malignancy and ranked as the fourth leading cause of cancer-related deaths worldwide [1]. Among all new liver cancer cases worldwide, the most common histological subtype is hepatocellular carcinoma (HCC), accounting for almost 70–85% [2]. The overall survival (OS) rate of HCC patients is poor (the 5-year OS rate is approximately 18%), despite opportunity for surgical resection [3]. Due to its high malignancy, insidious onset and rapid progression, HCC patients are usually in an advanced stage or have distant metastases at diagnosis, and these features are related to a poor prognosis [4]. Hepatitis B virus (HBV) infection is one of the critical factors in HCC prognosis, which causes the development of HCC [5] and produces resistance to sorafenib [6].

The World Health Organization declared that approximately 257,000,000 people are carriers of HBV [7], and HBV infection has gradually become a factor that cannot be ignored in HCC [8]. Worldwide, HBV carriers account for more than half of HCC cases [9, 10]. Studies have shown that HBV X protein (HBx), a viral protein composed by 154 amino acids and encoded by the HBV genome, plays an essential role in maintaining the replication of HBV [11, 12]. An early study showed that altered host gene expression by HBx caused the occurrence of HCC in transgenic mice [13]. The HBx protein regulates its target genes through epigenetic modification in the pathogenesis of HBV–HCC [14]. In a previous study, we confirmed that activation of key oncogenes by epigenetic modification is a key factor leading to the occurrence and development of HBV-related HCC [15]. However, the clinical significance of HBx proteins in the pathogenesis of HCC remains unclear.

Enhancers, a class of cis-regulatory elements, play a vital role in orchestrating spatiotemporally precise gene expression programs during epigenetic regulation. In 2013, the concept of super-enhancers (SEs) was first reported as a new type of gene regulatory element [16]. SEs are described as clusters of stretch enhancers with an abnormally high level of enrichment in combination with transcription factors and coactivators [17], and numerous gene expression disorders are regulated by SEs. SEs show unique histone modifications, including H3K27ac and H3K4me1, which can be easily identified by chromatin immunoprecipitation and deep sequencing (ChIP-seq) [18]. H3K27ac functions as a typical characteristic marker for identifying active enhancers, and researchers have identified SEs based on the abundance of H3K27ac in genomic DNA [19–21]. In SE-mediated regulation, extensive colocalization between transcription factors (TFs) and SEs was found as well as extensive cooperativity [22]. SEs are enriched for TFs, which drive gene expression and control cell identity [23, 24]. SEs recruit coactivators, including the mediator complex and p300, to alter chromatin accessibility, resulting in the interaction of enhancers, promoters or RNA polymerase with TFs [25]. Stimulus factors can reprogram the SE and mRNA landscape to influence cell development and heterogeneity [26]. Importantly, SEs can also activate oncogenes to promote the proliferation, invasion and migration of tumor cells [27, 28]. It will be of great interest to identify and characterize SE-related TFs in the pathogenesis of HCC that accompanies HBV infection.

The ETS transcription factor family plays a critical role in controlling cell homeostasis, including proliferation, differentiation, apoptosis, cell cycle, tissue remodeling, and angiogenesis [29]. ETV4 belongs to one of the 568 cancer-driven genes, which were revealed by carefully designed bioinformatics methods from approximately 28,000 tumor samples of 66 cancer types [30]. Overall, a disorder of downstream target genes expression has a crucial role in ETV4-driven tumors, and ETV4 target genes and signaling involved in the regulation of the potential cancer cells have been reported, including the activation of MMP1 [31, 32] and RAS/MAPK signaling [33]. Therefore, identifying other undefined obscure targets will provide a better understanding of the regulation mechanisms involved in HCC.

Disheveled family proteins (DVL1, DVL2, and DVL3) [34] function as critical components of the Wnt/β-catenin signaling pathway [35] and govern several cell biological processes, including invasion, migration, proliferation, and differentiation [36]. Activation of the canonical Wnt pathway is triggered by a coreceptor complex that contains Wnt proteins, the seven-pass transmembrane Frizzled (Fz) receptor and lipoprotein receptor related protein (LRP) 5/6 [37]. A previous study showed that DVL2 is regulated by GABARAPL1 and degrades through the autophagy pathway to inhibit Wnt signal transduction [38]. Further studies have shown that DVL2 can phosphorylate GSK3β to inhibit β-catenin degradation [39]. Another work proposed that the loss of SETD2 enhanced the mRNA and protein expression levels of DVL2, which led to the activation of the Wnt signaling pathway [40]. The variety of target genes activated by the Wnt/β-catenin signaling pathway are diverse and include c-MYC, MMP7, CCND1, CD44, LEF1, and c-JUN [41, 42]. These various mechanisms of β-catenin activation result in target gene expression, which eventually causes a distinct HCC phenotype.

## Materials and methods

### Cell lines and culture

The HCC cell lines Hep3B, HepG2, HepG2.2.15, SH-HEP-1, MHCC97H, and SMMC7721 as well as immortalized human L02 cells were purchased from the TCC Cell Bank (Shanghai, China). All cells were cultured in Dulbecco’s modified Eagle’s medium (DMEM, Wisent, 319–010-CL) or Eagle’s minimum essential medium (EMEM, Wisent, 320–006-CL) containing 10% fetal bovine serum (FBS, Wisent, 085–150) and penicillin–streptomycin liquid (Invitrogen, 15140–122). The cells were incubated (37 °C) in a humidified incubator with 5% CO_2_.

### HCC tissue samples

HCC tissues and surrounding nontumor tissues were collected from 40 HCC patients who received hepatic resection at Zhongda Hospital of Southeast University in China from 2013 to 2018. All patients were informed and agreed to participate in this epigenetics research. This analysis was approved by the Ethics Committee of Zhongda Hospital affiliated with Southeast University, China (Ethics Approval No. 2021ZDKYSB032).

### Gene expression profiles

Cultured L02 cells were constructed with pcDNA4.0-vector or pcDNA4.0-HBx using Lipofectamine 2000, and L02 cells were selected with G418. A total of 1 × 10^6^ cells were lysed in each sample with TRIzol Reagent, and total RNA was isolated. An Oligotex mRNA Mini Kit (Qiagen) was performed to extract mRNA from total RNA for reverse transcription (Invitrogen, 18080093) and subsequent RNA-seq library preparation (Guangzhou Epibiotek Co., Ltd., Guangzhou, China). After the RNA-seq library underwent quality inspection, it was sequenced using Illumina HiSeq 2500 and NextSeq 500, and general transcriptome sequencing data were obtained. The RNA-seq libraries were subjected to QC analysis and sequenced using Illumina HiSeq 2500 and NextSeq 500. The filtered reads were then mapped to the human reference genome HG19. The FPKM of each gene was obtained by HTSeq v0.9.1. The differentially expressed genes between pcDNA4.0-vector and pcDNA4.0-HBx (three biological replicates per group) were analyzed by the DESeq R package (version 1.18.0). The differentially expressed mRNAs were estimated according to an absolute fold change ≥ 1.5 and *P* < 0.05.

### Gene set enrichment analysis (GSEA)

GSEA (version 4.1.0) was performed by the Molecular Signatures Database (MSigDB) [43]. The correspondence between all genes and gene sets is available at MSigDB. The normalized enrichment score (NES) and false discovery rate (FDR) were used to calculate the significance score of GSEA.

### Analysis of The Cancer Genome Atlas (TCGA) and UALCAN databases

The data of HCC patients from TCGA were downloaded from TCGA data portal (http://tcga-data.nci.nih.gov/tcga/). Three hundred and sixty-five HCC patients were included in this study. Large-scale global gene expression profiling was processed and analyzed by R language, and the matrix data were log_2_-transformed. The target genes expression in HCC and corresponding adjacent tissues were compared by processed TCGA data. The prognostic value of genes was analyzed by TCGA data using the Kaplan–Meier model and log-rank test. The UALCAN database (http://ualcan.path.uab.edu/) was used to assess the relationship between the ETV4 expression level and the tumor stage, nodal metastasis status, tumor grade and promoter methylation level.

### Western blotting (WB)

Cells were lysed with RIPA lysis buffer and centrifuged at 12,000 rpm for 10 min at 4 °C to remove cell debris. The bicinchoninic acid (BCA) protein detection kit (Thermo, QL227061) was utilized for the quantitative determination of the protein concentration. The quantified protein was separated by 10% sodium dodecyl sulfate-polyacrylamide gel electrophoresis (SDS–PAGE) and transferred onto a 0.22 μm PVDF membrane (Sigma, 46978100). After blocking with 5% non-fat dry milk for 2 h at 26 °C, the membranes were incubated with anti-ETV4 (Abcam, ab189826), anti-DVL2 (CST, #3224), anti-H3 (CST, #4499S), anti-β-catenin (CST, #8480), non-phospho (Active) β-catenin (CST, #8814), anti-MMP7 (CST, #8655T), and anti-c-MYC (CST, #8655T), with anti-β-actin (Sigma, QC45480) as reference gene. After incubation with HRP-conjugated secondary antibody (CST, #7074 #7076), a chemiluminescence scanner (Tanon) and ImageJ software were used for analysis.

### Quantitative real-time PCR (qRT–PCR) analysis

Total RNA was isolated from cultured cells or tissue samples with TRIzol reagent (Invitrogen, 252703), and total RNA was reverse transcribed with a reverse transcription kit (Takara, AK71648A). qRT-PCR was performed on samples with SYBR Green (Takara, AK41789A) and a StepOne Plus system (Applied Biosystems, Foster City, CA) in triplicate. The target genes expression was normalized by β-actin using the 2^−ΔΔCt^ calculation method. Supplementary Table S1 lists all of the primer sequences used for qRT–PCR.

### Vector construction, lentiviral construction, and cell transfection

The 3rd generation lentiviral packaging vectors (lentivirus vector GV492/493, pHelper 1.0 and pHelper 2.0) and Lipofectamine 2000 (Invitrogen, #2307486) transfection reagent were used to simultaneously transfect HEK293T cells to obtain lentiviral particles (GeneChem Co., Ltd., Shanghai, China). The cells were resuspended in 100 mm cell culture dishes at 80% density with 10% serum medium, and lentiviral particles along with HitransG A or HitransG P were mixed in the culture medium to infect the cells. Stable ETV4 overexpression or knockdown cells were generated by puromycin (2–5 µg/ml) selection.

### Cell migration and invasion and wound healing assays

Cell migration and invasion were examined by the Transwell (Costar, USA, #3422) migration assay. In the Transwell migration assay, HCC cells were precultured in serum-free medium for 48 h, 3.5 × 10^4^ cells were seeded in the upper chamber with serum-free medium, and the lower chambers were filled with DMEM containing 10% FBS. For the Transwell invasion assay, the Matrigel (BD, USA, #SPC-356234) was spread on the bottom of the upper chamber before the cells were seeded. After 48 h, the unmigrated cells were removed with cotton swabs, and the migrated cells were fixed, stained and counted using an IX71 inverted microscope (Olympus, Tokyo, Japan). In the wound healing assay, HCC cells were digested into individual cells and plated on 6-well plates at 3 × 10^5^ cells per well. A pipette tip free of bacteria was used to scrape the HCC cell monolayer, and the cell migration changes were assessed by microscopy at 0, 24, and 48 h after scraping. It is declared that no blinding was done in animal studies.

### Animal experiments

Twelve female nude mice approximately 4–6 weeks old were randomly divided into two groups. A total of 2 × 10^6^ MHCC97H-LV-shControl and MHCC97H-LV-shETV4 cells were injected into nude mice through the tail vein. After injection, the mice continued to be reared, and mouse body weights were measured every 6 days. Thirty days after rearing, the nude mice were euthanized. The lung and liver tissues of nude mice were dissected, and the number of metastatic tumors was counted. The animal study was approved by the Ethics Committee of Zhongda Hospital affiliated with Southeast University and all procedures followed the guidelines of the Medical School of Southeast University.

### Chromatin immunoprecipitation (ChIP) and ChIP-qPCR assay

ChIP experiments were performed using the SimpleChIP® Plus Sonication Chromatin IP Kit (CST, Kit #56383) according to the manufacturer’s instructions. In short, 1 × 10^7^ cells in each sample were fixed with formaldehyde to cross-link DNA proteins. The chromatin was sheared using Bioruptor® Pico (Diagenode Co., Ltd., Belgium) (11 cycles of sonication: 30 s on, 30 s off, total running time 11 min), and then the sheared chromatin (10 µg) was incubated with antibodies (1 μl of IgG, 1 μl of H3K27ac, 2 μl of ETV4, and 10 μl of histone H3), along with ChIP-Grade Protein G magnetic beads (30 μl). Antibodies used in the ChIP assay included normal rabbit IgG (Cell Signaling Technology, #2729), ETV4 (Abcam, ab189826), H3K27ac (Abcam, ab4729) and histone H3 rabbit mAb (Cell Signaling Technology, #4620). Then, the protein-DNA cross-linking was reversed, and the DNA was separated and purified. The enrichment of DNA sequences was detected using qPCR, and the primers are shown in Tables S2 and S3. Each experiment was performed in triplicate.

### Luciferase assay

The pGL3-Basic and pRL-TK vectors constituted a dual-luciferase reporter system. The plasmid was purchased from the GENEWIZ Co. Ltd. Suzhou, China. In brief, we cloned an approximately 1267 bp region of the DVL2 promoter and its deletion fragments into a pGL3-Basic vector expressing the firefly luciferase reporter gene. Empty pGL3-Basic vector was transferred as a systemic control. Hep3B and HepG2.2.15 cells were cultured with a density of 2 × 10^5^ cells per well and plated in 12-well plates before transfection. The cells were transfected with 760 ng of the DVL2 promoter-driven luciferase plasmid(s), 760 ng of the pcDNA3.1-ETV4 plasmid and 76 ng of the pRL-TK plasmid through transfection reagent (Lipofectamine 2000). The Renilla luciferase generated by the pRL-TK plasmid was considered as an internal control to evaluate the transfection efficiency in cells. After 36 h of transfection, the cells were harvested. The luciferase reporter gene assay kit was used to lyse and react with the cells in the dark room. Then, the firefly and Renilla luciferase activity was determined. The signal of luciferase activity was normalized and exhibited as the relative luciferase activity. Each set of transfection data was acquired in triplicate.

### Statistical analysis

Independent Student’s *t*-test was used to compare the results, and the error bars represent the mean ± SD. The log rank test was used in the prognostic analysis to verify the significance of differences between the two groups. To determine the correlation between two variables, Pearson’s correlation coefficient was calculated, and a *P* value less than 0.05 indicated a significant difference. (*P* < 0.05, *P* < 0.01, *P* < 0.001; ns represents not significant).

## Results

### Altered gene expression profile in L02-HBx and L02-vector cells

**Fig. 1:**
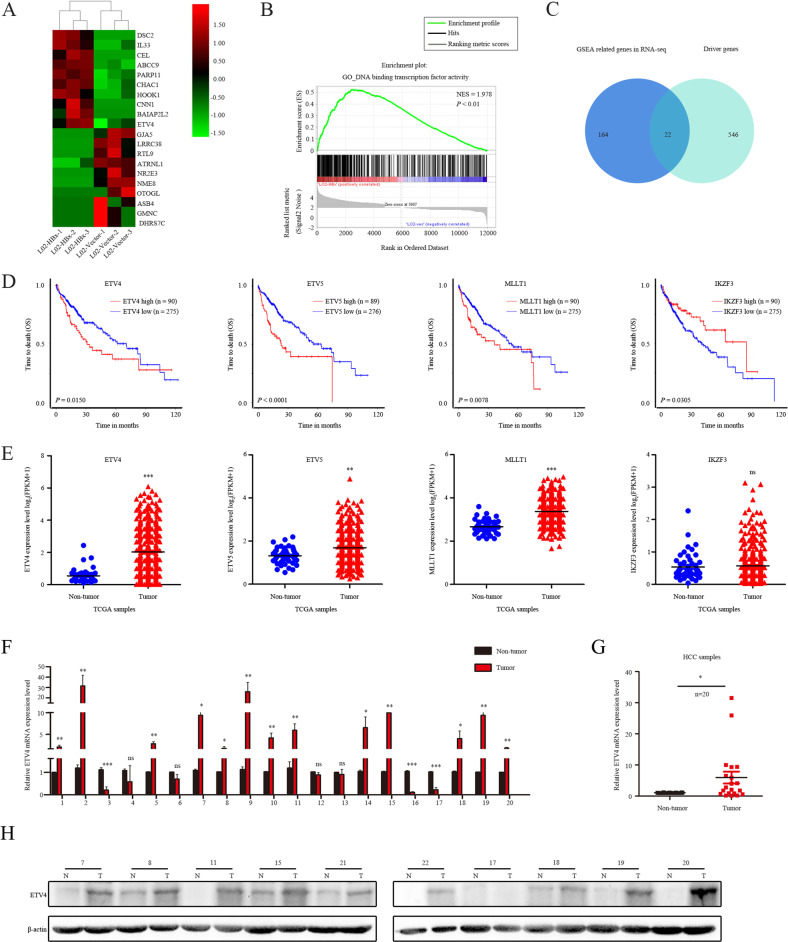
Altered gene expression profile in L02-HBx and L02-vector cells. **A** Heatmap of differentially expressed genes (DEGs) induced by HBx transfection in the RNA-seq analysis. **B** Gene set enrichment analysis (GSEA) comparing the gene sets of DNA binding TF activity in the HBx group. NES represents the normalized enrichment score (*P* < 0.01). **C** Twenty-two genes were identified after intersection analysis between the DNA binding TF activity signaling pathway and 568 driver genes using Venn diagram software. **D** Kaplan–Meier survival curve analysis representing probabilities of overall survival (OS) in 371 HCC patients according to ETV4, ETV5, MLLT1, and IKZF3 expression. Statistical analysis was conducted using Student’s *t*-test and the log-rank test. **E** Four of 22 intersectional genes expression showed significant differences in 371 HCC tissues and 50 nontumor tissues from TCGA database. **F** The mRNA level of ETV4 was detected by qRT–PCR in 20 pairs of HCC tissues. **G** The statistical graph shows the mRNA expression levels of ETV4 in 20 pairs of HCC tissues. **H** ETV4 protein level was detected by Western blotting (WB) in ten pairs of HCC tissues.

To gain better insights into the mechanism of HBx-induced HCC, RNA-seq analysis was performed to profile the gene expression patterns of L02-HBx and L02-vector cells, and the raw data has been uploaded (GSE 186862). Our data revealed that 5905 genes were upregulated in L02-HBx cells, while 6077 genes were downregulated (absolute fold-change cutoff >1.5, *P* < 0.05). The heatmap indicated the top ten differentially expressed genes (DEGs) in L02-HBx and L02-vector cells (Fig. 1A). GSEA, the pathway enrichment method for evaluating microarray data at the gene set level, was applied to reveal the mechanism of HBx. We performed GSEA with the raw expression to compared the differentially expressed pathways caused by HBx versus vector. GSEA of HBx-related RNA-seq data demonstrated that the DEGs were highly enriched in the DNA binding TF activity gene set (NES = 1.978, *P* < 0.01) (Fig. 1B). The intersection of the DNA binding TF activity gene set and the 568 tumor driver genes [30] revealed 22 TFs that play an important role in HCC (Fig. 1C). To estimate the diagnostic and prognostic values of these 22 TFs, the original data of 371 HCC cases were downloaded from TCGA (https://tcga-data.nci.nih.gov/docs/publications/). Kaplan–Meier analysis and the log rank test were used to compare the survival curves for the 22 genes, and the results verified that high expression of only four (ETV4, ETV5, MLLT1, and IKZF3) of the 22 genes was significantly associated with poor overall survival (OS) in HCC (Fig. 1D). The other 18 genes expression showed no significant association with OS (Supplementary Fig. S1A–E). Furthermore, the expression patterns of the four mentioned genes were depicted in HCC patients from TCGA compared with corresponding nontumor subjects (Fig. 1E). Among them, ETV4 expression showed the most significant changes in the two groups. To investigate the role of ETV4 in the development of HCC, we further explored expression patterns of ETV4 in HCC tissue samples and adjacent nontumor tissues and found mRNA levels of ETV4 to be significantly elevated in tumors compared with matched adjacent normal tissues (Fig. 1F). The specific primers used are shown in Supplementary Table S1. Based on the mRNA levels of ETV4, ETV4 was frequently increased in 65% (13/20) of HCC patients (Fig. 1G and Supplementary Fig. S1F). To validate these results, we explored expression of ETV4 protein in ten matched pairs of HCC and corresponding nontumor tissues by Western blotting (WB). Similarly, the WB results verified that ETV4 protein expression was upregulated in HCC tissues compared with nontumor tissues (Fig. 1H). These data implied that ETV4 is upregulated in HCC and that the expression pattern is closely related to a poor prognosis for HCC patients.

### ETV4 is a candidate SE-associated gene upregulated by HBx through chromatin accessibility

**Fig. 2:**
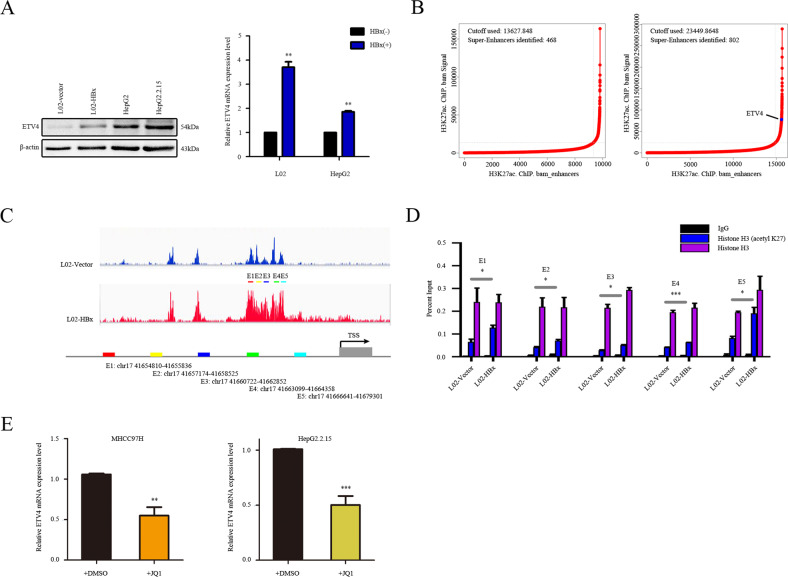
ETV4 is a candidate SE-associated gene upregulated by HBx through chromatin accessibility. **A** WB and qRT-PCR to detect ETV4 level in L02-HBx cells and its control (*P* < 0.001); WB and qRT-PCR to detect ETV4 expression level in HepG2 cells and HepG2.2.15 cells (*P* < 0.001). **B** Hockey stick plots showing normalized and rank-ordered H3K27ac signals for SE-associated genes in L02-HBx cells and the corresponding control cells. **C** The SE region of ETV4 was divided into five constituent enhancer regions (E1–E5) to design primers for the ChIP-qPCR assay. **D** ChIP-qPCR assay with ETV4 antibody to confirm the direct interaction between ETV4 and its potential target gene DVL2 promoter region in L02-HBx and L02-vector cells. **E** JQ1 (inhibitor of BRD4, one major component of SE) treatment inhibited ETV4 transcription in L02-HBx cells and L02-vector cells.

Our collective analyses suggest that ETV4 dysregulation plays a crucial role in the initiation and progression of HCC. Then, we confirmed the regulation of ETV4 by HBx in L02-vector, L02-HBx, HepG2, and HepG2.215 cells. We found that ETV4 expression were upregulated at the mRNA and protein levels by HBx (*P* < 0.01; Fig. 2A). In addition, we found that the ETV4 expression significantly correlated with the HCC stage, lymph node metastasis, grade, and promoter methylation level according to TCGA database analysis with UALCAN (Supplementary Fig. S2A–D).

As ETV4 has been acknowledged as a master TF that drives PD-L1L2-SE, genetic silencing or pharmacological inhibition in cancer cells [44], we investigated whether ETV4 has a similar SE mechanism in HCC. We applied the core regulatory circuitry (CRC)_Mapper [45] to H3K27ac ChIP-seq data from L02-HBx and L02-vector cells and identified 427 SEs, which were then subjected to rank ordering of super-enhancers (ROSE) [16, 46] analysis to identify whether ETV4 is related to SEs (Fig. 2B). Genome-wide studies have confirmed that SEs can be defined as DNA sequences that bind to H3K27ac and other epigenetic methods, including H3K4me1 [47, 48]. Our H3K27ac ChIP-seq results demonstrated that HBx could reprogram the *cis*-regulatory landscape by inducing de novo SEs. The human ETV4 genomic locus with the ChIP-Seq database for H3K27ac is shown in Supplementary Fig. S2E. According to the ChIP-seq data, we identified an H3K27ac-enriched region considered to be a SE located approximately 10 kb upstream of the ETV4 transcriptional start site (TSS) (Supplementary Fig. S2E).

HBx can directly impact chromatin and transcription [49]. Moreover, H3K27ac is considered to be an active/open chromatin marker [50]. To verify the L02-HBx and L02-vector ChIP-seq data and further investigate whether the chromatin accessibility changes induced by HBx are attributed to the upregulation of ETV4, we performed a H3K27ac ChIP-qPCR assay. The potential SE region of ETV4 was further divided into five constituent enhancers (E1–E5) and was further incorporated into a pGL3-promoter vector for the dual-luciferase reporter assay. Five pairs of specific ChIP-qPCR primers were designed for the five constituent enhancer fragments (E1-E5) (Fig. 2C and Supplementary Table S2). Using ChIP-qPCR, we validated that the binding of H3K27ac modifications in the ETV4 enhancer was significantly enhanced after HBx introduction (Fig. 2D). JQ1, an inhibitor of BRD4, is reported to repress transcriptional coactivation effects and downregulate expression of SE-specific genes [46, 51]. THZ1, an inhibitor of CDK7, which is considered to be a SE-targeting drug, showed mild antiproliferative effects compared with JQ1 [52, 53]. We validated whether inhibitors of BRD4 and CDK7 have the ability to destroy the SE-specific ETV4 expression. BRD4 (JQ1, 0.5 μM) and CDK7 (THZ1, 10 nM) were added to MHCC97H and HepG2.2.15 cells for 48 hours. The results demonstrated that BRD4 exposure significantly reduced ETV4 expression in both MHCC97H and HepG2.2.15 cells (Fig. 2E), while the change in ETV4 was no significant after THZ1 exposure (Supplementary Fig. S2F). These data indicate that ETV4, which is upregulated by HBx, is an SE-associated gene related to BRD4.

### ETV4 facilitates HCC cell migration and invasion in vitro and in vivo

WB and qRT–PCR analysis showed that ETV4 was overexpressed in HCC cell lines (HepG2, HepG2.2.15, SK-HEP-1, Hep3B, MHCC97H, and SMMC7721) compared with its expression in the control (L02) (Supplementary Fig. S3A). The dysregulation of ETV4 in HCC tissues and cell lines inspired us to further explore its oncogenic function in HCC. ETV4-overexpressing HepG2 and SK-HEP-1 cells were generated and displayed relatively low endogenous ETV4 expression (Supplementary Fig. S3B, C). Similarly, ETV4-knockdown HepG2.2.15 and MHCC97H cells were produced and displayed relatively high endogenous ETV4 expression (Supplementary Fig. S3D, E). GSEA was performed using GSE101728 datasets and HCC-related functional gene sets derived from MSigDB. The results confirmed that ETV4 expression mainly affected the adherens junction pathway in HCC (Supplementary Fig. S3F).

**Fig. 3:**
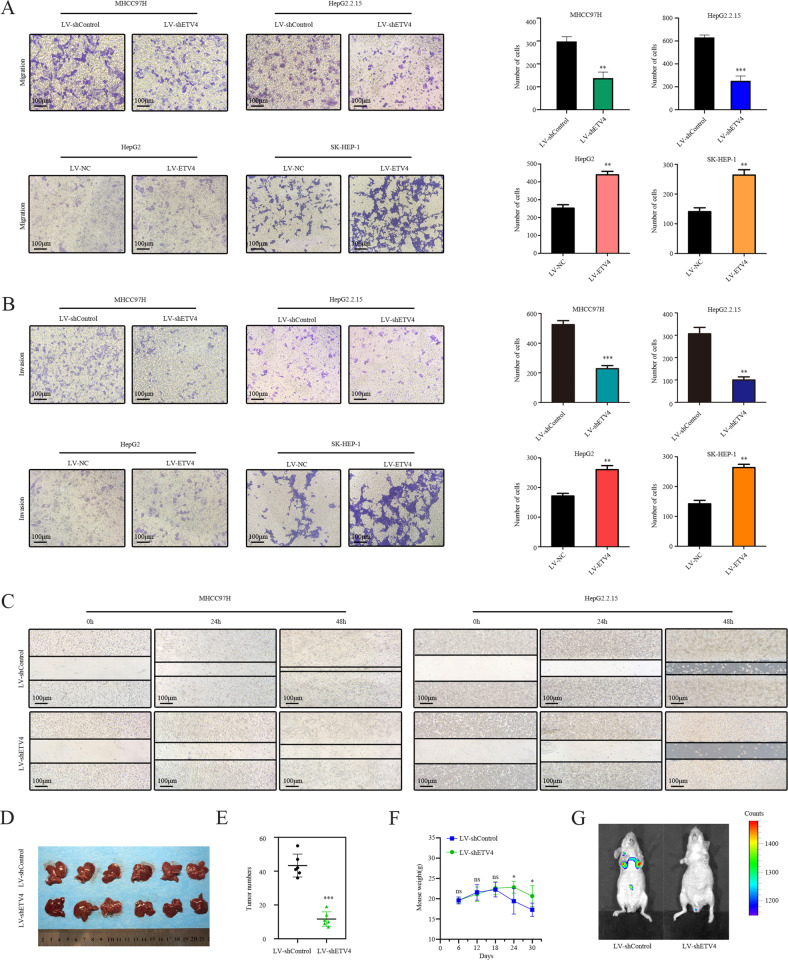
ETV4 facilitates HCC cell migration and invasion in vitro and in vivo. **A** Transwell migration assays for MHCC97H and HepG2.2.15 or HepG2 and SK-HEP-1 cells with stable ETV4 knockdown or overexpression (*P* < 0.01; *P* < 0.001; *P* < 0.01; *P* < 0.01). **B** Transwell invasion assays for MHCC97H and HepG2.2.15 or HepG2 and SK-HEP-1 cells with stable ETV4 knockdown or overexpression (*P* < 0.001; *P* < 0.01; *P* < 0.01; *P* < 0.01). **C** Wound healing assay. Wound closure was delayed in stable ETV4 knockdown cells compared with control cells at 0, 24, and 48 h. **D** In vivo, tumor transfer was examined by injecting MHCC97H-LV-shControl (left) or MHCC97H-LV-shETV4 (right) cells into the tail veins of nude mice. The number of tumor lesions on the liver was counted after resection from the mice. **E** The statistical graph shows the number of tumor lesions on the liver in nude mice injected with stable ETV4 knockdown cells and control cells. **F** The body weight of mice in both groups was recorded every 6 days. **G** Representative lung metastasis images obtained by an IVIS in vivo imaging system after inoculation.

ETV4 knockdown significantly reduced the migration and invasion of HCC cells, while ETV4 overexpression enhanced the migration and invasion of HCC cells (Fig. 3A, B). The wound healing assay was employed to confirm the function of ETV4 in promoting wound healing. The results revealed a significant reduction in wound healing ability after stable knockdown of ETV4 (Fig. 3C). To further verify the role of ETV4 in promoting HCC metastasis in vivo, we injected stable ETV4 knockdown MHCC97H cells into the lateral tail veins of nude mice. The animal experiments confirmed that the liver metastases and lower mouse weights were reduced in the ETV4 knockdown group compared with its control group (Fig. 3D–F). The nude mouse images obtained by an IVIS in vivo imaging station indicated that the stable ETV4 knockdown group had fewer lung metastases than its control group (Fig. 3G). These data demonstrated that ETV4 facilitates HCC cell migration and invasion functions in vivo and in vitro.

### ETV4 transcriptionally regulates DVL2

**Fig. 4:**
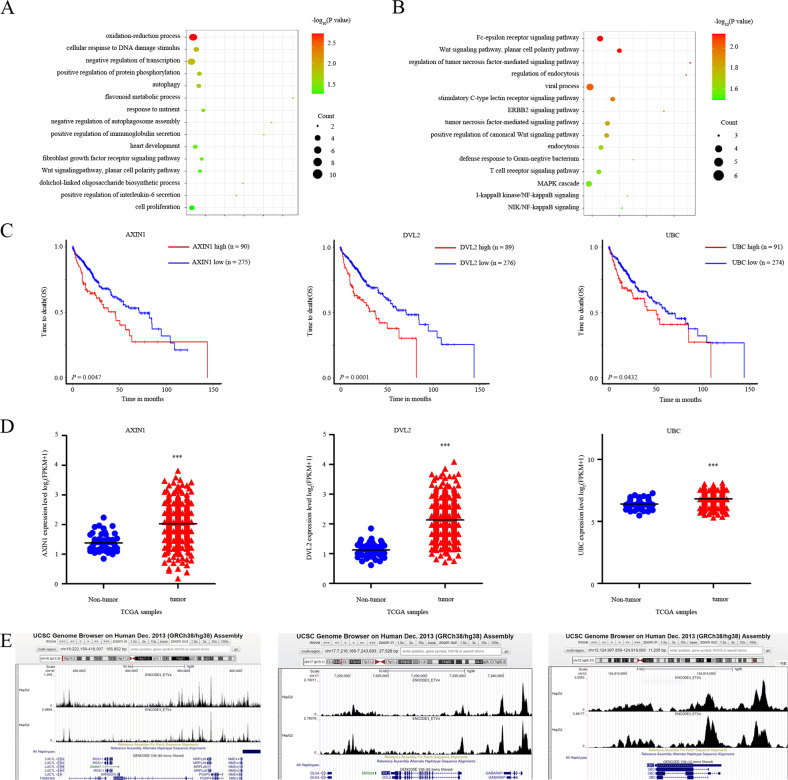
ETV4 transcriptionally regulates DVL2. **A**, **B** The analysis of ETV4 ChIP-seq data is available on the Cistrome Data Browser platform. The scores of possible ETV4 binding molecules were assessed from the ChIP-seq data, and the top 100 genes were subjected to gene ontology (GO) analysis. **C** Kaplan–Meier survival curve analysis according to three of seven genes in the Wnt/β-catenin pathway showed that high expression levels of AXIN, DVL2, and UBC correlated with worse overall survival. **D** The AXIN, DVL2, and UBC expression were higher in tumor tissues than in nontumor tissues (*P* < 0.005; *P* < 0.001; *P* < 0.05). **E** Batch sample visualization through the Wash U browser showing the cobinding pattern between ETV4 and the promoter regions of AXIN, DVL2, and UBC in HepG2 cells.

As a member of the ETS transcription factor family, ETV4 is a key regulatory protein that can change the hundreds of genes expression in various cancers. ChIP-seq experiments were conducted to profile the binding of TFs to DNA at a genome-wide scale. We explored all published ETV4 ChIP-seq profiles available in the Cistrome Data Browser (DB) (cistrome.org/db), and finally, 2 ChIP-seq profiles related to HCC were included. These ChIP-seq datasets provide the top 100 genes ranked by score, and then gene ontology (GO) analysis was performed. The GO analysis results revealed that target genes were mainly enriched in the Wnt signaling pathway (Fig. 4A, B). In HBV-HCC, the Wnt signaling pathway induces changes in epithelial–mesenchymal transition (EMT) [54] and proliferation [55]. The seven genes in the Wnt signaling pathway obtained from GO analysis were subjected to Kaplan–Meier analysis. The results indicated that the OS of HCC patients with high AXIN1, DVL2, and UBC expression was significantly worser than that of HCC patients with low expression (Fig. 4C). The other four genes expression had no significant correlation with HCC prognosis (Supplementary Fig. S4A). AXIN1, DVL2, and UBC had higher expression levels in HCC tissues than in nontumor tissues (Fig. 4D). Interestingly, the published ChIP-seq data from Cistrome DB showed that ETV4 can bind to the promoters of AXIN1, DVL2 and UBC via the University of California Santa Cruz (UCSC) Genome Browser (Fig. 4E). Among the scores of ETV4 binding to the three gene promoters, DVL2 ranked the highest. We next analyzed the association between DLV2 expression and the clinical characteristics of HCC patients by UALCAN, including HCC stage, lymph node metastasis, grade, and promoter methylation level. As shown, ETV4 expression correlated significantly with all of these clinical characteristics (Supplementary Fig. S4B).

### DVL2 is required for ETV4-mediated HCC metastasis and invasion

**Fig. 5:**
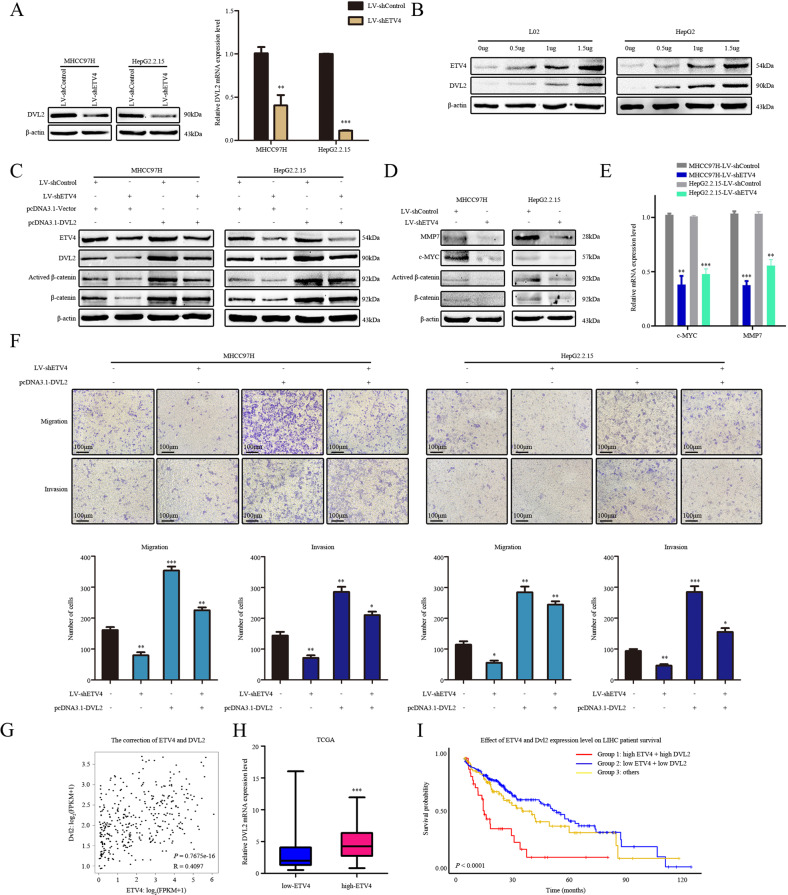
DVL2 is required for ETV4-mediated HCC metastasis and invasion. **A** In MHCC97H and HepG2.2.15 cells, ETV4 was stably knocked down, and DVL2 expression levels were detected by WB and quantitative real-time PCR (qRT–PCR) analysis. **B** In MHCC97H and HepG2.2.15 cells, the DVL2 expression level was detected by WB after transfection with the pcDNA3.1-ETV4 plasmid at different doses. **C** After the DVL2 construct was transfected into MHCC97H-LV-shETV4 and HepG2.2.15-LV-shETV4 cells, WB was performed to detect the rescue of the indicated protein expression. **D** WB assay showed that ETV4 regulate the protein expression levels of c-Myc and MMP7 by active β-catenin. **E** qPCR assays showed that ETV4 regulated c-MYC and MMP7 in transcriptional level. **F** In vitro, the migration and invasion capacity of MHCC97H and HepG2.2.15 cells were observed by knocking down ETV4 and then rescuing DVL2 expression. **G** Pearson’s correlation coefficient was conducted to evaluate the correlation between ETV4 and DVL2 mRNA level. The scatter plots showed the correlation of *R* = 0.4097 and *P* < 0.0001. **H** The positive relationship between the ETV4 and DVL2 expression was exhibited in 140 HBV-positive HCC patients. **I** The patients were divided into three groups as follows: group 1: high ETV4 + high DVL2, group 2: low ETV4 + low DVL2, and group 3: others. The combined prognosis analysis of ETV4 and DVL2 showed that group 1 had worse overall survival than the other two groups of HCC patients (*P* < 0.001).

We further validated that ETV4 mediates HCC migration and invasion by regulating DVL2. We knocked down ETV4 in HCC cells and found that the mRNA and protein levels of DVL2 were both decreased (Fig. 5A). In addition, increasing amounts of ETV4-expressing plasmid caused a significant dose-dependent accumulation of DVL2 proteins in L02 and HepG2 cells (Fig. 5B). The WB results indicated that overexpression of DVL2 prominently restored the decrease in β-catenin expression in ETV4 knockdown MHCC97H and HepG2.2.15 cells (Fig. 5C). In addition, we found that ETV4 influenced expression of β-catenin and active β-catenin, along with their target molecules, including MMP7 and c-MYC (Fig. 5D). To verify the mechanism, the qPCR results in MHCC97H and HepG2.2.15 cells showed that knockdown of ETV4 significantly reduced the mRNA expression of c-MYC and MMP7 (Fig. 5E). Simultaneously, the migration and invasion assay indicated that overexpression of DVL2 rescued the cell migration and invasion ability induced by knockdown of ETV4 in vitro (Fig. 5F). These data demonstrated that the regulation of β-catenin by ETV4 is DVL2 dependent.

Expression of ETV4 and DVL2 was first evaluated by TCGA database analysis. We found that the positive correlation between ETV4 and DVL2 mRNA expression was exhibited in HCC patients (*R* = 0.4097) (Fig. 5G). To further explore the correlation between ETV4 and DVL2 in HBV–HCC tissues, we analyzed a total of 140 HCC samples that were positive for hepatitis B surface antigen (HBsAg) among the 371 HCC samples. We divided the HCC samples according to the cutoff value into a high ETV4 expression group and a low ETV4 expression group, and the results implied that ETV4 and DVL2 expression correlated positively in HBV–HCC (Fig. 5H). To better explore the effect of these genes on the survival of HCC patients, a combination analysis of ETV4 and DVL2 was performed. Patients with higher levels of both ETV4 and DVL2 had the shortest survival time, and patients with lower levels of both ETV4 and DVL2 had the longest survival time (Fig. 5I). Collectively, ETV4 and DVL2 expression can indicate unfavorable prognosis in HCC patients.

### ETV4 upregulates DVL2 expression and its transcriptional activity by directly binding to the DVL2 promoter

**Fig. 6:**
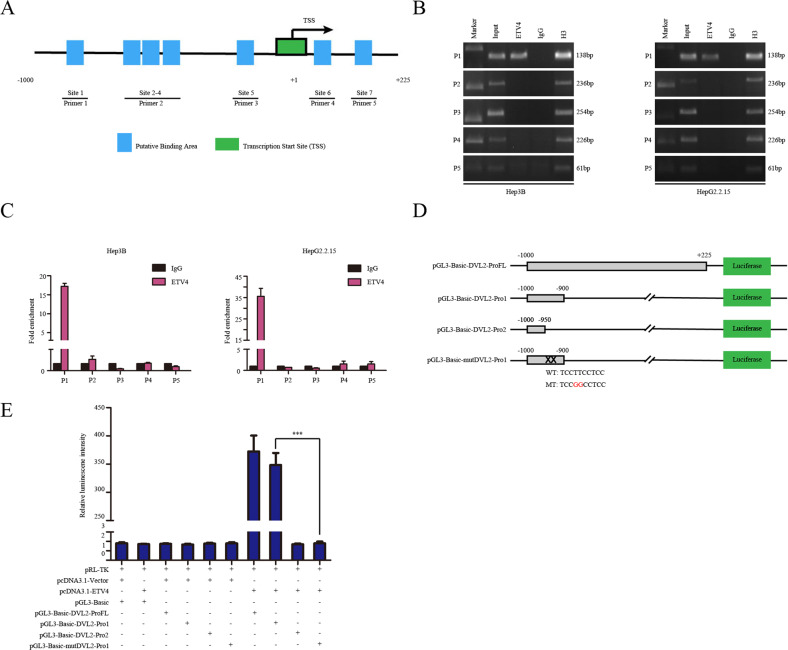
ETV4 upregulates DVL2 expression and its transcriptional activity by directly binding to the DVL2 promoter. **A** A schematic representation of the ChIP-PCR and ChIP-qPCR primers designed around the DVL2 promoter region. Seven predicted binding sites were designed to be included in five pairs of primers. **B** ChIP-PCR assay with ETV4 antibody to validate the direct interaction between ETV4 and the DVL2 promoter region in Hep3B and HepG2.2.15 cells. The results emphasize that ETV4 can bind at site 1 (−918 bp to −909 bp) of the DVL2 promoter region. Cell lysates before precipitation were removed separately as input (1%). IgG was a negative control provided by the kit. H3 was a positive control provided by the kit. **C** ChIP-qPCR assay with ETV4 antibody to validate the direct interaction between ETV4 and the DVL2 promoter region in Hep3B and HepG2.2.15 cells. The results confirm that ETV4 can bind at site 1 (−918 bp to −909 bp) of the DVL2 promoter region. IgG was a negative control provided by the kit. **D** Schematic diagram of the construction of a dual luciferase reporter gene construct. The DVL2 promoter fragment was inserted into the pGL3-Basic plasmid. ProFL represents the full length of the DVL2 promoter region, Pro1 represents −1000 bp to −900 bp, Pro2 represents −1000 bp to −950 bp, and mutPro1 indicates that Pro1 has been mutated. **E** The double luciferase reporter gene experiment showed that ETV4 combined with DVL2 promoter site 1. When predicted binding site 1 was mutated, the luciferase activity was rescued.

Through the JASPAR database and UCSC genome website, we obtained the sequence of the DVL2 promoter and predicted seven potential ETV4 binding sites (Supplementary Fig. S4C). To explore whether ETV4 directly binds to the DVL2 promoter in vitro, ChIP-PCR and ChIP-qPCR assays were conducted. As shown, the putative seven binding sites (−1000 to +225) were covered by five pairs of primers (Fig. 6A and Supplementary Table S3). We performed ChIP-PCR and ChIP-qPCR assays in Hep3B and HepG2.2.15 cells and found that site 1 (−936 to −946) was the unique ETV4 binding site in the DVL2 promoter (Fig. 6B, C). For further verification, the full length (a 1225-bp region 5′ to the transcriptional start site), truncated fragments and mutated constructs of the DVL2 promoter were cloned into a luciferase reporter plasmid (PGL3-Basic) (Fig. 6D). Then, we transfected the ETV4-overexpressing plasmid, corresponding empty plasmid, luciferase reporter vector pGL3-Basic plasmid and Renilla luciferase reporter plasmid pRL-TK vector into Hep3B cells. The luciferase activity results revealed that the overexpression of ETV4 significantly enhanced the transcriptional activity of reporter plasmids of full-length reporter P-1000/+225-luc and promoter1 reporter P-1000/-900-luc compared with the pcDNA3.1 group (Fig. 6E). No significant change was observed in the shorter DVL2 promoter 2 reporter P-1000/−900-luc group (Fig. 6E). After cotransfection of the mutated constructs along with the ETV4 overexpression plasmid, the mutated reporter P-1000mut/−900-luc had a suppression effect on DVL2 promoter activity compared with the full-length P-1000/+225-luc group and promoter1 P-1000/-900-luc group (Fig. 6E). Overall, these data suggested that ETV4 regulates DVL2 expression in transcriptional level and that the −936 to −946 region of the DVL2 promoter is critical for ETV4-mediated DVL2 transcription.

## Discussion

Metastatic liver cancer remains a major challenge worldwide for patients, their families and clinicians. Surgical resection is the best treatment for primary liver cancer, while metastatic liver cancer has a poor prognosis due to its high metastatic potential [56, 57]. In the process of tumor cell metastasis, abnormal changes (activation or inactivation) in oncogenes and tumor suppressor genes are commonly recognized mechanisms [58]. The molecular mechanisms underlying HCC metastasis are very complicated and remain largely unknown.

HBV is a primary cause of acute and chronic viral hepatitis and further leads to the occurrence of HCC. Increasing evidence indicates that HBx is involved in the regulation of HCC proliferation, invasion, migration, and glucose metabolism [59, 60]. Furthermore, in vivo experiments have shown that HBx, acting as a transactivating factor of viral genes, may change various genes expression and lead to the occurrence of HCC [13]. HBx interacts with cellular proteins to activate various signaling pathways or affect gene expression by epigenetic regulation [61, 62]. Studies have reported that the protein levels of HDAC1 are upregulated by HBx [63, 64], but it is still unclear whether the active enhancer or SE is associated with H3K27ac. Our study detected the redistribution of H3K27ac enrichment after overexpression of HBx, and we analyzed the chromatin accessibility of the ETV4-associated SE region. The SE regulation mode was strongly associated with the regulation of SE-related genes [19, 65]. This further reveals that HBx activates ETV4 and potentially other oncogenes through an SE-associated mechanism. Additionally, we reported that HBx upregulates ETV4 at the mRNA and protein levels to promote HCC progression for the first time.

ETV4 belongs to the ETS transcription factor family, which is one of the largest evolutionarily conserved transcription factor families; members of this family have a unique feature: the conserved DNA-binding ETS domain [66]. It was reported that the aberrant expression of ETV4 plays a critical role in the pathogenesis of various malignancies, including breast cancer, lung cancer, endometrial cancer, gastric cancer, and HCC [31, 67–70]. Studies in HCC have shown that ETV4 can regulate cell proliferation, invasion and migration and can also increase sorafenib sensitivity and promote cisplatin resistance [31, 71, 72]. Previous studies have suggested that ETV4 plays a role in affecting tumor cell migration and invasion through a colony-forming assay by regulating MMP1 [31]. There is still little known about the underlying mechanism related to ETV4. The activation of ETV4 plays a critical role in the metastasis and invasion of HBV-related HCC, but the correlation between ETV4 and HBx is not clear. Our results indicate that HBx regulates ETV4 through an SE-associated mechanism and that HBx partially regulates chromatin accessibility. We first proposed that ETV4 can regulate MMP7 to influence the migration and invasion function of HCC cells.

Wnt/β-catenin signaling is often observed to be dysregulated in HCC [73]. Activated β-catenin accumulates in the cytoplasm, then transfers to the nucleus, and forms a nuclear complex with TCF/LEF factors (such as TCF4) for transcriptional regulation [74]. Once the Wnt signaling pathway is activated, its downstream genes expression is active, including β-catenin, TCF4, ZEB2, c-MYC, MMP7, and CCND1 [75, 76]. Recent studies have demonstrated that DVL2 could activate Wnt/β-catenin signaling [77]. In this study, we demonstrated that ETV4 could bind to the promoter of DVL2, which consequently activated Wnt/β-catenin signaling and its target genes related to migration and invasion.

**Fig. 7:**
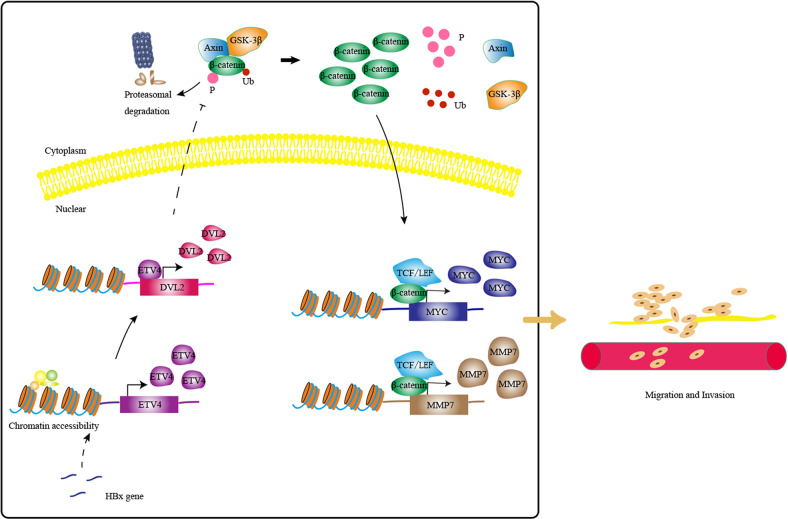
A schematic diagram showing that the ETV4 functions in HCC metastasis. HBx induces the oncogenic effects of ETV4 in HCC by regulating DVL2 in transcriptional level, and then influence the Wnt/β-catenin signaling pathway to promote HCC metastasis and invasion.

In summary (as shown in Fig. 7), we demonstrated that overexpression of ETV4 facilitates cell metastasis of HCC in vitro and in vivo. The ETV4 expression is related to tumor grade, stage, lymph node metastasis and prognosis in HCC patients. ETV4 activates DVL2 by directly binding to its promoter region, thereby inhibiting degradation of the β-catenin protein. Therefore, targeting the ETV4-DVL2-β-catenin axis provide novel mechanistic insights and therapeutic strategy for HCC.

## Supplementary information

aj-checklist

Supplementary Fig. S1

Supplementary Fig. S2

Supplementary Fig. S3

Supplementary Fig. S4

Supplementary Figure legend

Supplementary Table. S1

Supplementary Table. S2

Supplementary Table. S3

Editing Certificate

## Data Availability

The datasets used and/or analyzed during the current study are available from the corresponding author on reasonable request.
