# Supplementary figures and images for: HBx increases chromatin accessibility and ETV4 expression to regulate dishevelled-2 and promote HCC progression
Source: Cell Death Dis. 2022 Feb 4;13(2):116. doi: 10.1038/s41419-022-04563-9 (PMC8816937; doi:10.1038/s41419-022-04563-9)

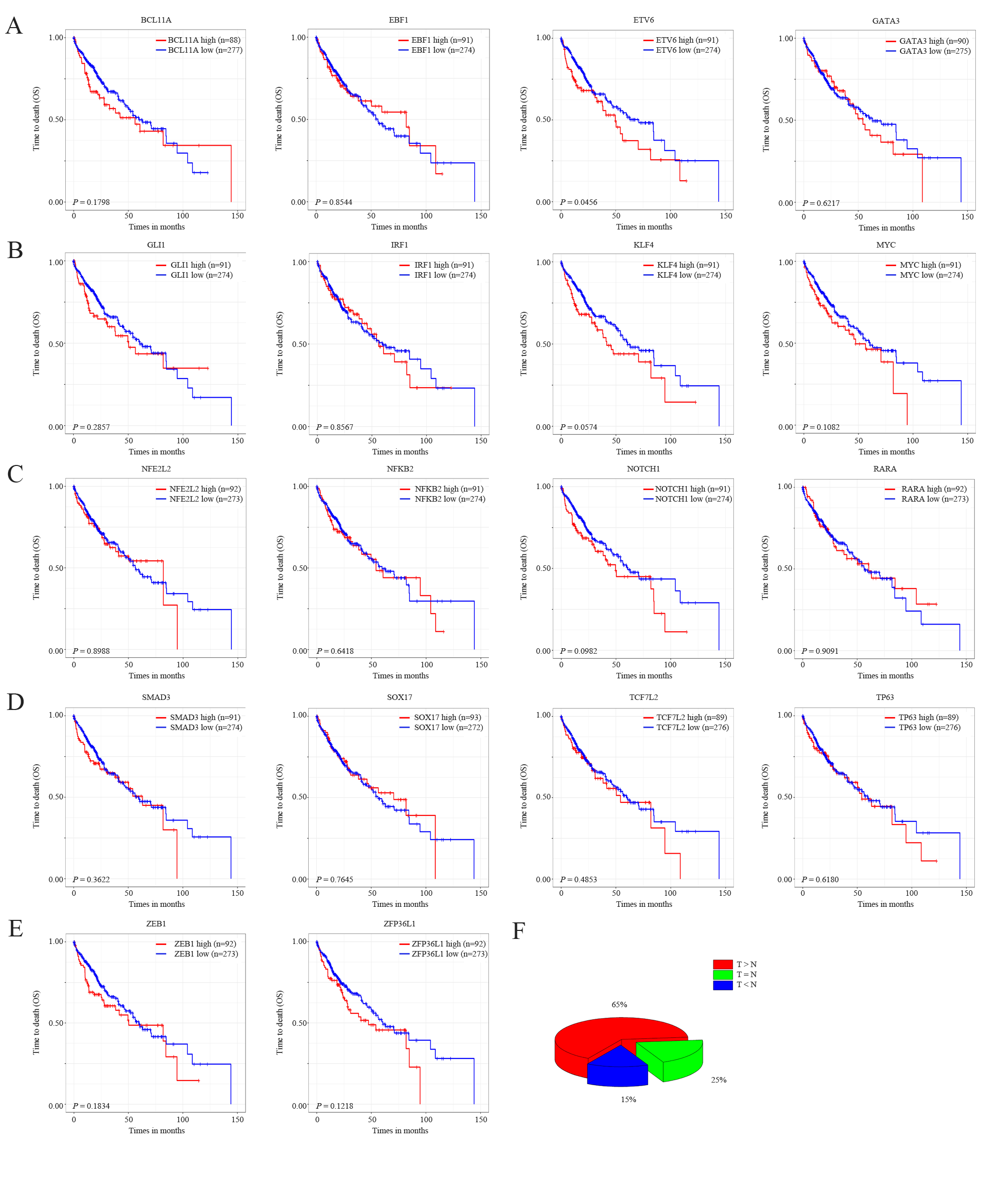

Supplement: Supplementary file 2 — Supplementary Fig. S1 [file 41419_2022_4563_MOESM2_ESM.png]

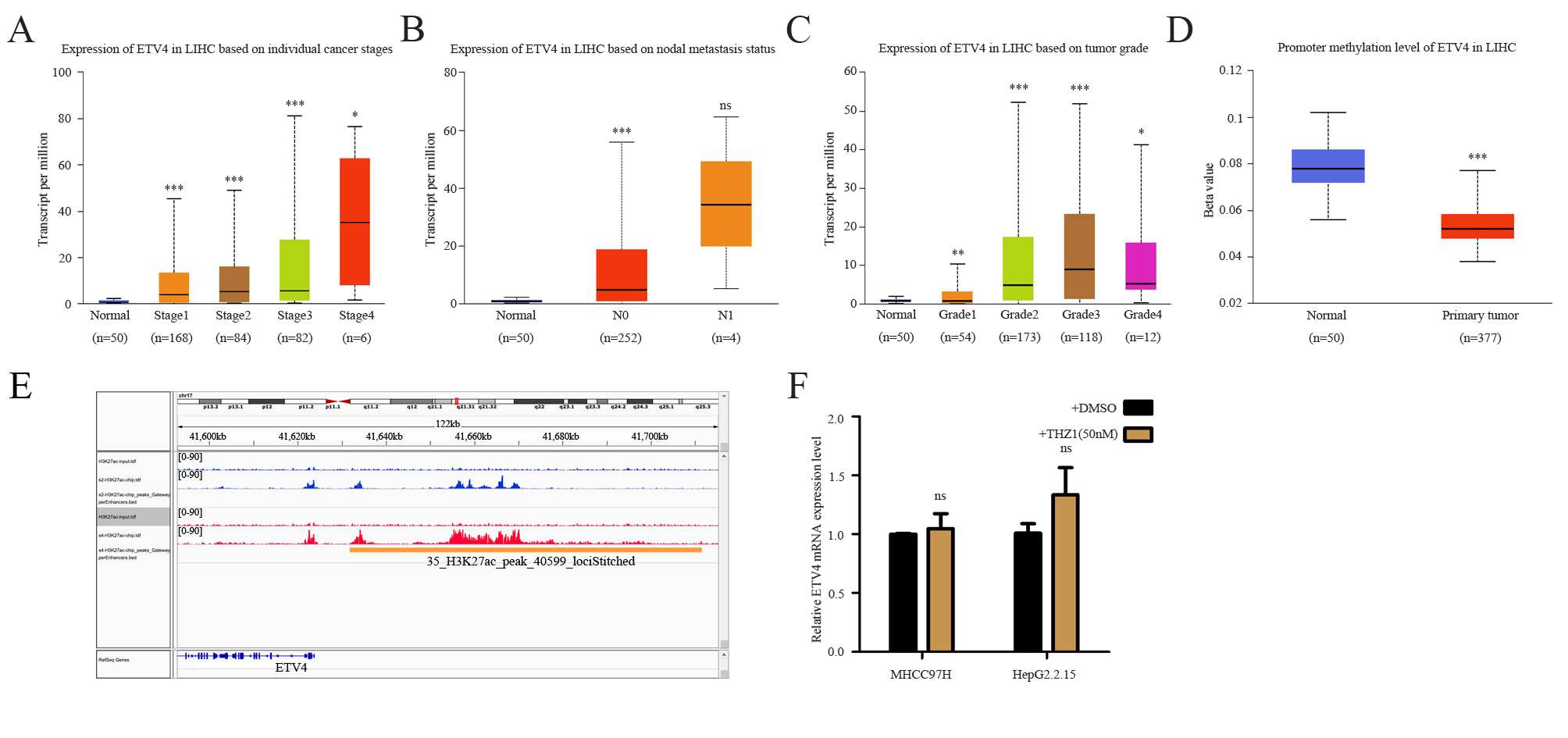

Supplement: Supplementary file 3 — Supplementary Fig. S2 [file 41419_2022_4563_MOESM3_ESM.png]

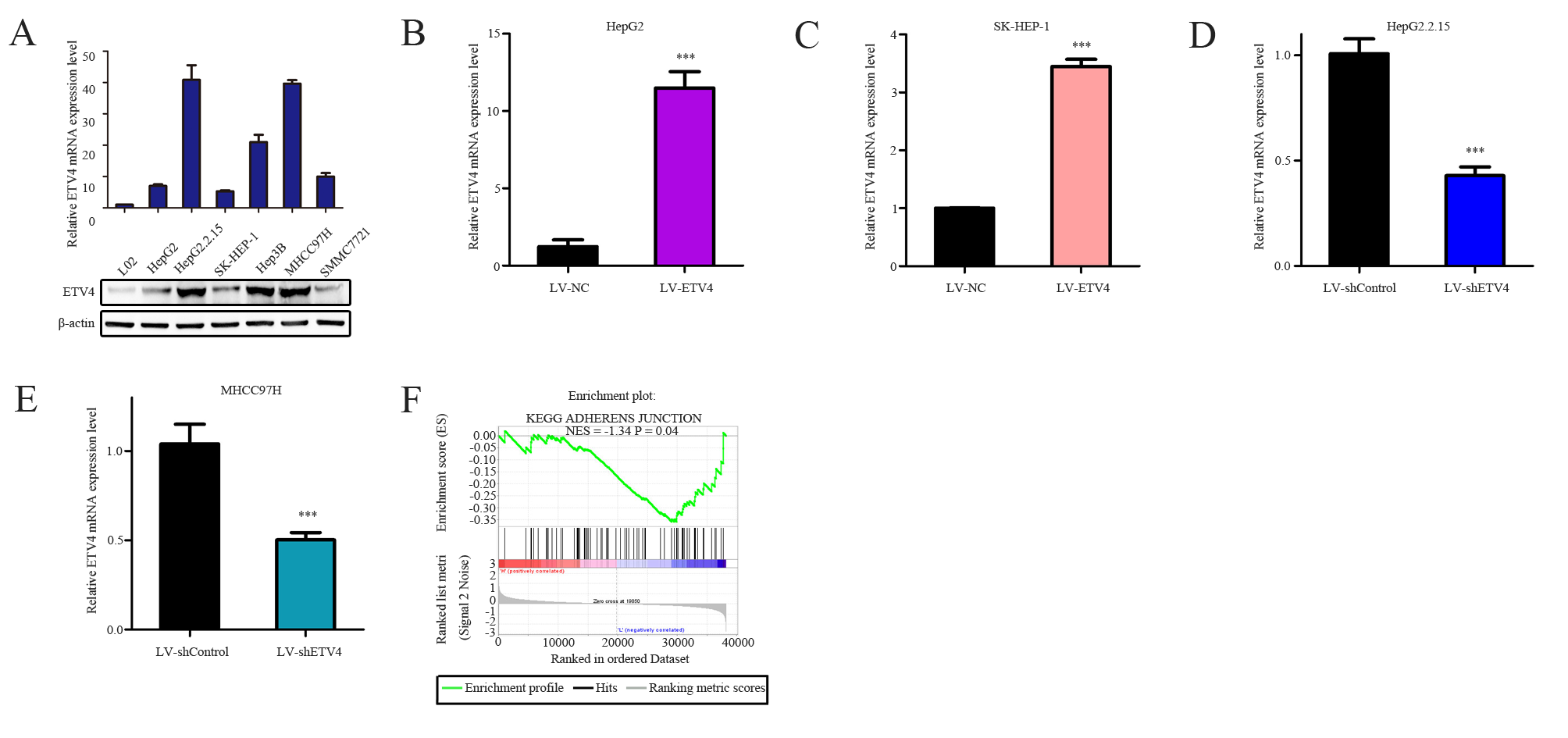

Supplement: Supplementary file 4 — Supplementary Fig. S3 [file 41419_2022_4563_MOESM4_ESM.png]

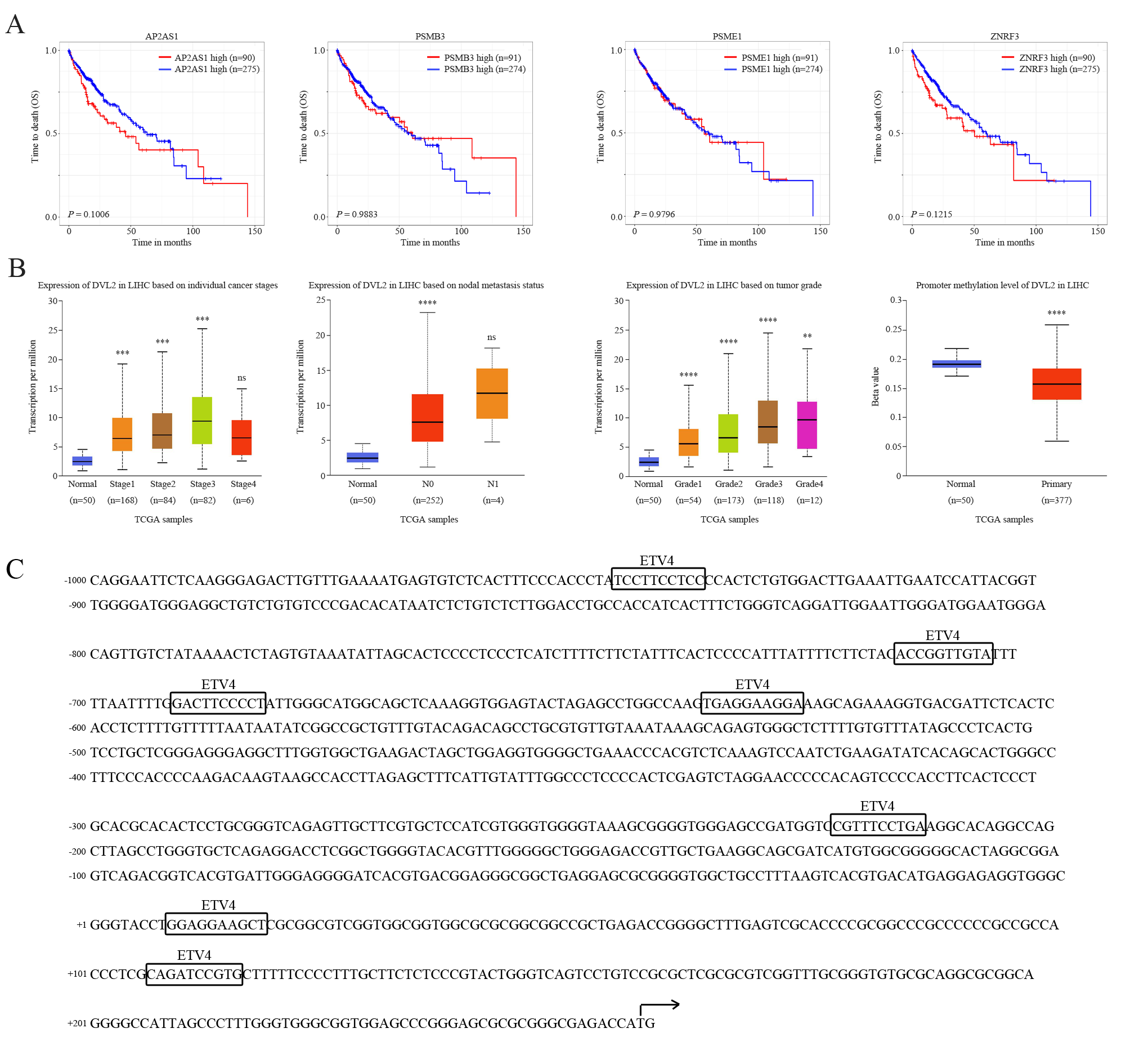

Supplement: Supplementary file 5 — Supplementary Fig. S4 [file 41419_2022_4563_MOESM5_ESM.png]
