# Supplementary material for: HBx increases chromatin accessibility and ETV4 expression to regulate dishevelled-2 and promote HCC progression
Source: Cell Death Dis. 2022 Feb 4;13(2):116. doi: 10.1038/s41419-022-04563-9 (PMC8816937; doi:10.1038/s41419-022-04563-9)
Supplement: Supplementary file 6 — Supplementary Figure legend [file 41419_2022_4563_MOESM6_ESM.docx]

Figure S1. The 18 overlapping genes were subjected to prognostic analysis with raw TCGA data.

1. -E. Kaplan–Meier plots showing the association between the expression of 18 genes and overall survival.

F. The pie chart shows the statistical results of 20 pairs of HCC tissues with high and low expression of ETV4.

Figure S2. The relationship between the ETV4 expression level and clinical information in the UALCAN database and its related SE mechanism.

A. Expression of ETV4 in the liver hepatocellular carcinoma (LIHC) cohort based on individual cancer stages (*P* < 0.05; *P* < 0.001).

B. Expression of ETV4 in the LIHC cohort based on the nodal metastasis status (*P* < 0.001).

C. Expression of ETV4 in the LIHC cohort based on tumor grade (*P* < 0.05; *P* < 0.01; *P* < 0.001).

D. Promoter methylation levels of ETV4 in the LIHC cohort (*P* < 0.001).

E. Based on a snapshot of chromatin immunoprecipitation (ChIP)-seq data, in L02-HBx cells, the enhancer region of ETV4 showed much more H3K27ac enrichment than its control.

F. Treatment with the SE inhibitor THZ1 had no effect on ETV4 transcription in MHCC97H and HepG2.2.15 cells.

Figure S3. Screening of HCC cell lines and construction of stably transfected cell lines.

A. WB and qRT–PCR analysis of ETV4 expression in a human nonmalignant cell line and HCC cell lines.

B & C. HepG2 and SK-HEP-1 cells with stable overexpression of ETV4 were constructed (*P* < 0.001 and *P* < 0.001, respectively).

D & E. HepG2.2.15 and MHCC97H cells with stable knockdown of ETV4 were constructed (*P* < 0.001 and *P* < 0.001, respectively).

F. GSEA of ETV4 based on the GSE101728 datasets showed that ETV4 expression was positively correlated with the adherens junction signaling pathway.

Figure S4. Prognostic analysis of 4 Wnt/β-catenin pathway genes with the TCGA database and assessment of the relationship between Dvl2 and clinical characteristics.

A. Kaplan–Meier survival curves for 365 HCC patients showed that high levels of AP2AS1, PSMB3, PMSE1 and ZNRF3 expression were related to a poor prognosis (*P* = 0.1006; *P* = 0.9883; *P* = 0.9796; *P* = 0.1215).

B. Expression of Dvl2 in the LIHC cohort based on individual cancer stages, nodal metastasis status, and tumor grade (*P* < 0.01; *P* < 0.001; *P* < 0.0001).

C. Diagram of possible binding sites at which ETV4 binds to the DVL2 promoter region.
