# Supplementary material for: HBx increases chromatin accessibility and ETV4 expression to regulate dishevelled-2 and promote HCC progression
Source: Cell Death Dis. 2022 Feb 4;13(2):116. doi: 10.1038/s41419-022-04563-9 (PMC8816937; doi:10.1038/s41419-022-04563-9)
Supplement: Supplementary file 7 — Supplementary Table. S1 [file 41419_2022_4563_MOESM7_ESM.docx]

Table S1. List of primers used in qRT-PCR assays.

| Gene | Primer | Sequence |
| --- | --- | --- |
| ETV4 | Forword | 5’-CCCTGAGAAATTTGAAGGAGAC-3’ |
|  | Reverse | 5’-GTCCAGGCAATGAAATGGGCA-3’ |
| DVL2 | Forword | 5’-CCACCTTTACCTCCTTTGCCA-3’ |
|  | Reverse | 5’-CCATGCTCACTGCTGTCTCTCC-3’ |
| c-MYC | Forword | TACAACACCCGAGCAAGGAC |
|  | Reverse | TTCTCCTCCTCGTCGCAGTA |
| MMP7 | Forword | TACCCATTTGATGGGCCAGG |
|  | Reverse | AGACTGCTACCATCCGTCCA |
| β-actin | Forword | 5’-AAGGATTCCTATGTGGGCGAC-3’ |
|  | Reverse | 5’-CGTACAGGGATAGCACAGCC-3’ |
