# Supplementary material for: HBx increases chromatin accessibility and ETV4 expression to regulate dishevelled-2 and promote HCC progression
Source: Cell Death Dis. 2022 Feb 4;13(2):116. doi: 10.1038/s41419-022-04563-9 (PMC8816937; doi:10.1038/s41419-022-04563-9)
Supplement: Supplementary file 8 — Supplementary Table. S2 [file 41419_2022_4563_MOESM8_ESM.docx]

Table S2. List of primers used in ChIP-PCR and ChIP-qPCR with ETV4 antibody.

| Name | Sequence | Product |
| --- | --- | --- |
| Primer1-Forword | 5’-CCTCAGGGTCCTCAGCCTAT-3’ | 138bp |
| Primer1- Reverse | 5’-GACACAGACAGCCTCCCATC-3’ |  |
| Primer2-Forword | 5’-TTAGCACTCCCCTCCCTCAT-3’ | 236bp |
| Primer2- Reverse | 5’-GCAGGACAGTGAGGGCTATAA-3’ |  |
| Primer3-Forword | 5’-ATTGTATTTGGCCCTCCCCA-3’ | 254bp |
| Primer3- Reverse | 5’-CGTGATCCCCTCCCAATCAC-3’ |  |
| Primer4-Forword | 5’-TTGCTGAAGGCAGCGATCAT-3’ | 226bp |
| Primer4- Reverse | 5’-GGGAAAAAGCACGGATCTGC-3’ |  |
| Primer5-Forword | 5’-CTGAGACCGGGGCTTTGAG-3’ | 61bp |
| Primer5- Reverse | 5’-TGACCCAGTACGGGAGAGAA-3’ |  |
