# Supplementary material for: HBx increases chromatin accessibility and ETV4 expression to regulate dishevelled-2 and promote HCC progression
Source: Cell Death Dis. 2022 Feb 4;13(2):116. doi: 10.1038/s41419-022-04563-9 (PMC8816937; doi:10.1038/s41419-022-04563-9)
Supplement: Supplementary file 9 — Supplementary Table. S3 [file 41419_2022_4563_MOESM9_ESM.docx]

Table S3. List of primers used in ChIP-qPCR with H3K27ac antibody.

| Name | Sequence | Region |
| --- | --- | --- |
| E1-Forword | 5’-GCCTCACAGTCAAAGGTGCT-3’ | Chr17:41654810-41655836 |
| E1- Reverse | 5’-GTAACCGAGGCAAACACCCA-3’ |  |
| E2-Forword | 5’-AAGCTGCAAGGTGGCAAAAC-3’ | Chr17:41657174-41658525 |
| E2- Reverse | 5’-GTGCAAAGCGAGAAGATCCG-3’ |  |
| E3-Forword | 5’-GTAGGGGTCAAGTCCCCACT-3’ | Chr17:41660722-41662852 |
| E3- Reverse | 5’-GGAGTACCTGCTGGGCTAAG-3’ |  |
| E4-Forword | 5’-ACATATGGGCAGCGACTTGA-3’ | Chr17:41663099-41664358 |
| E4- Reverse | 5’-CTTTGCAGATCCACCCAGCA-3’ |  |
| E5-Forword | 5’-CTCTTCCCCAGCCCTAAAGC-3’ | Chr17:41666641-41667931 |
| E5- Reverse | 5’-ACCCGGCATTTCAGGTTCAA-3’ |  |
