# Supplementary material for: HBx increases chromatin accessibility and ETV4 expression to regulate dishevelled-2 and promote HCC progression
Source: Cell Death Dis. 2022 Feb 4;13(2):116. doi: 10.1038/s41419-022-04563-9 (PMC8816937; doi:10.1038/s41419-022-04563-9)
Supplement: Supplementary file 10 — Editing Certificate [file 41419_2022_4563_MOESM10_ESM.pdf]

AJE®

Editing Certificate

This document certifies that the manuscript

HBx increases chromatin accessibility and ETV4 expression to regulate dishevelled-2 and promote HCC progression

prepared by the authors

Chuqian Zheng, Min Liu, Yanping Ge, Yanyan Qian, Hong Fan

was edited for proper English language, grammar, punctuation, spelling, and overall style by one or more of the highly qualified native English speaking editors at AJE.

This certificate was issued on **December 3, 2021** and may be verified on the AJE website using the verification code **3C4F-3C85-89DF-14B4-C92P**.

Neither the research content nor the authors' intentions were altered in any way during the editing process. Documents receiving this certification should be English-ready for publication; however, the author has the ability to accept or reject our suggestions and changes. To verify the final AJE edited version, please visit our verification page at aje.com/certificate. If you have any questions or concerns about this edited document, please contact AJE at support@aje.com.

AJE provides a range of editing, translation, and manuscript services for researchers and publishers around the world.
For more information about our company, services, and partner discounts, please visit aje.com.
